# Dental Materials

**Published:** 1863-03

**Authors:** 


DENTAL MATERIALS.

The profession can, perhaps, no where obtain dental supplies better than of Sutton & Raynor, 782 Broadway, N. Y. They keep a full supply of every thing in the dental line, and at most reasonable rates. None need hesitate in ordering goods, for they are prompt, and their experience extensive. We feel confident that all orders filled by them will be satisfactory. And of course our brethren who visit New York will call upon Sutton & Raynor. *It will pay.* T.

